# Supplementary material for: Asian dust-storm activity dominated by Chinese dynasty changes since 2000 BP
Source: Nat Commun. 2020 Feb 20;11:992. doi: 10.1038/s41467-020-14765-4 (PMC7033097; doi:10.1038/s41467-020-14765-4)
Supplement: Supplementary file 1 — Supplementary Information [file 41467_2020_14765_MOESM1_ESM.pdf]

Supplementary Information

Asian dust-storm activity dominated by Chinese dynasty changes since 2000 BP

Chen et al.

Supplementary Figures

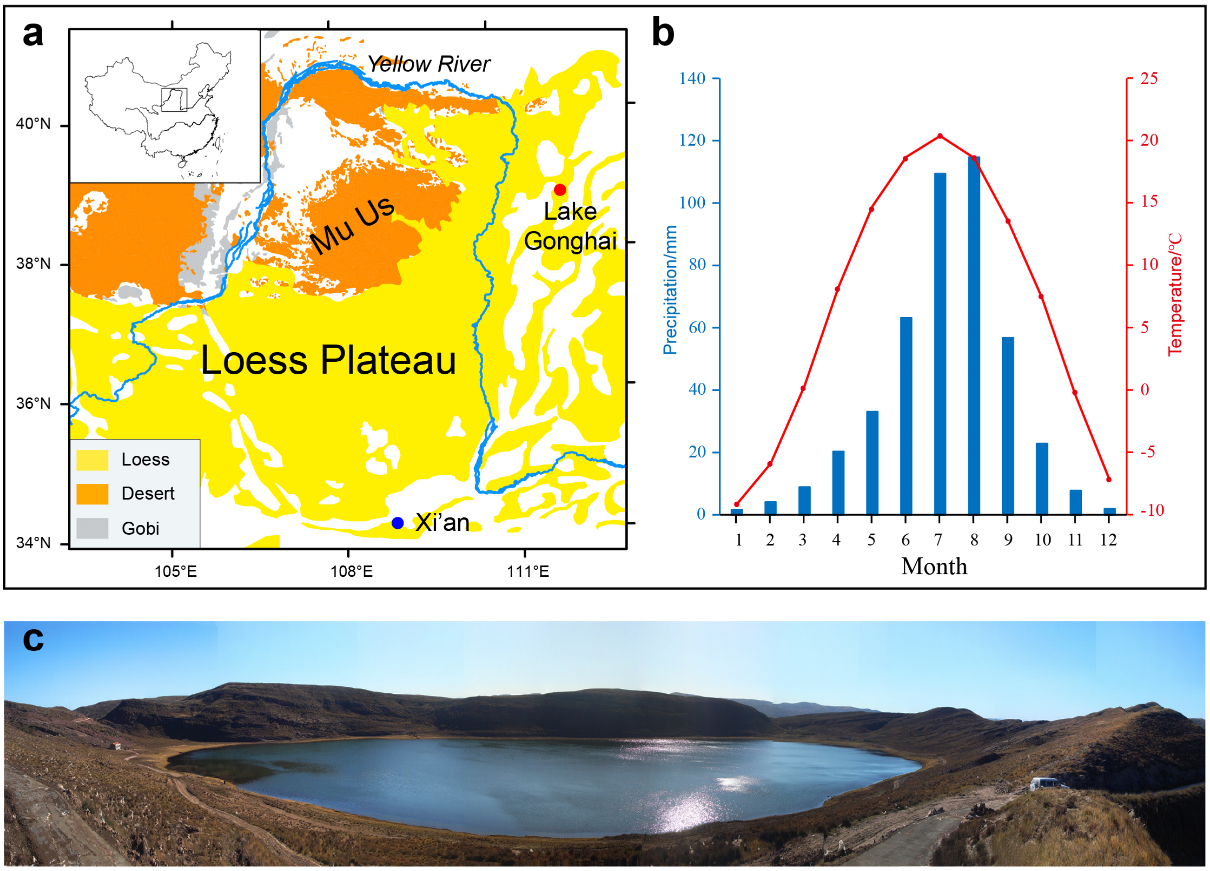

Supplementary Figure 1. Location and settings. a, Location of Lake Gonghai and the Mu Us Desert. Xi'an, the capital of six dynasties, is located in the southern margin of the Chinese Loess Plateau. **b**, Instrumental data of precipitation, temperature and wind speed (1981-2010) from nearby Ningwu Station, Shanxi, China (http://data.cma.cn). **c**, Panoramic view of Lake Gonghai. The Supplementary Fig. 1a was created using Arcmap 10.2.

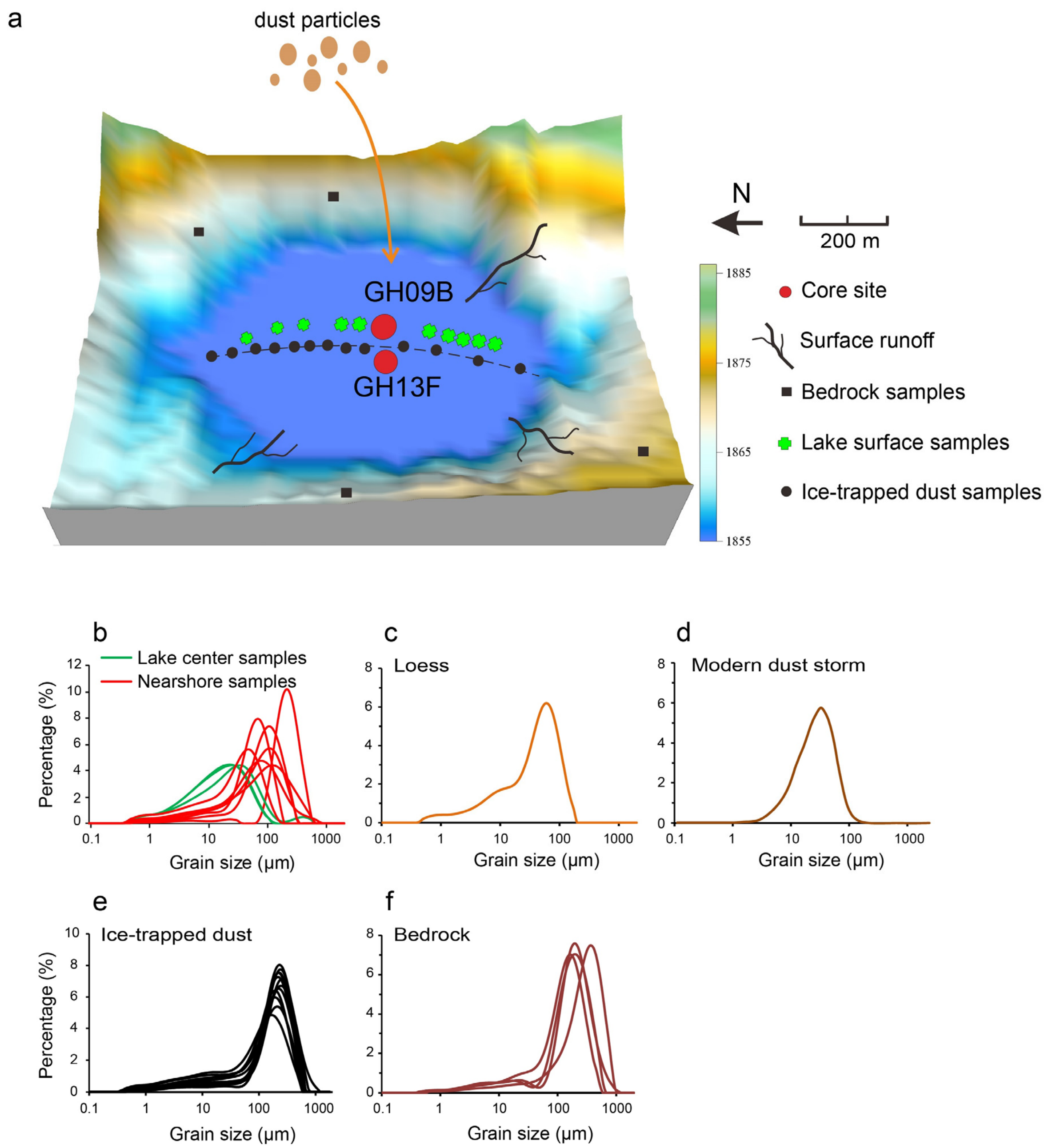

Supplementary Figure 2. Basin topography, lake sediment provenance, and grain-size distributions of various modern sediment types. a, Schematic map illustrating potential lake sediment sources. The sampling sites are indicated. **b-f**, Grain-size distributions of surface lake sediments (**b**), surrounding loess (**c**), modern dust storm deposition on the Chinese Loess Plateau[1] (**d**), ice-trapped dust (**e**) and bedrock from the lake catchment (**f**). The schematic map of the lake basin is based on altitude data measured by GPS and was created using Surfer 15.

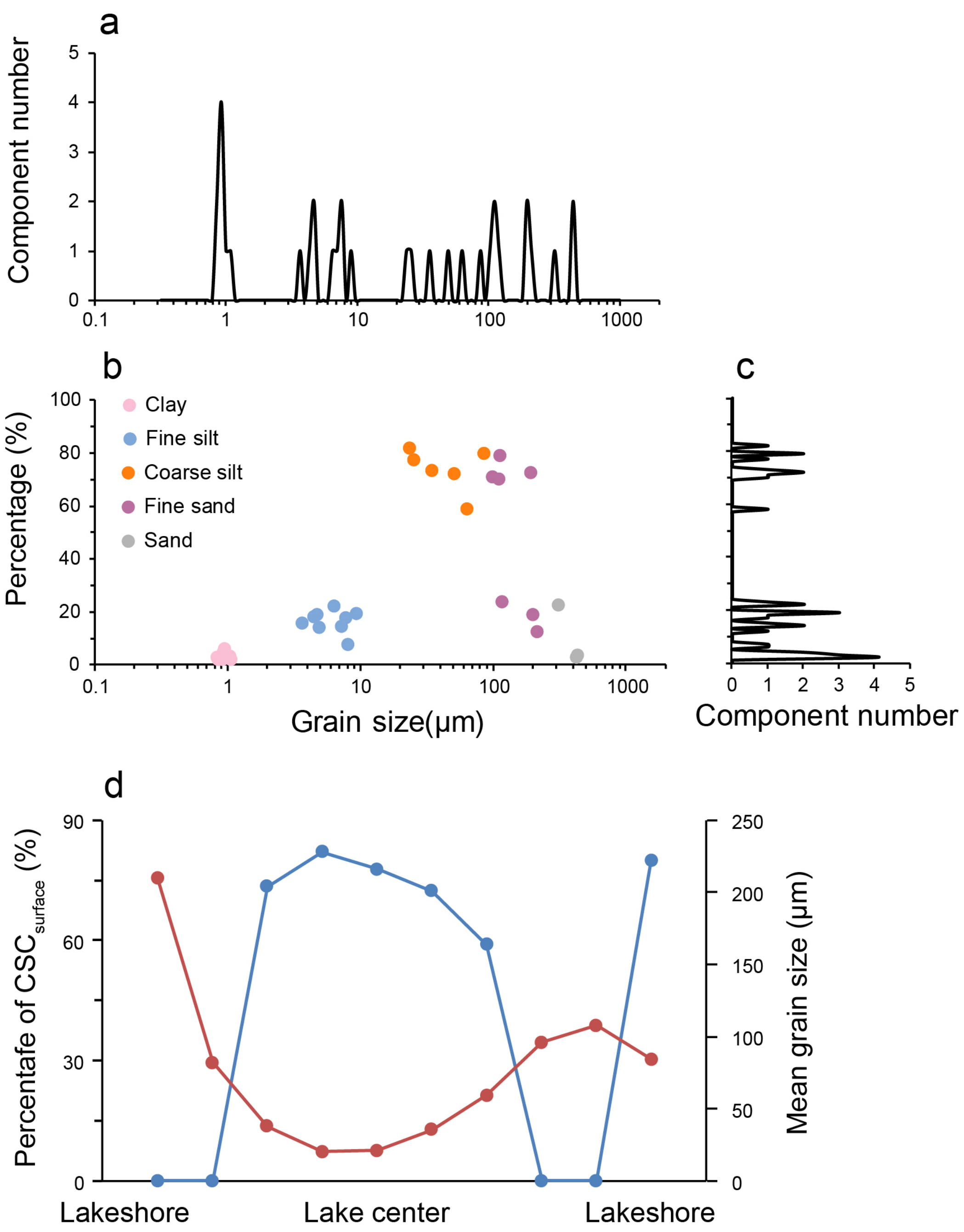

Supplementary Figure 3. Grain-size analysis results for surface lake sediments. a-c, Frequency of modal sizes (**a**), size-proportion plot of all grain-size components (**b**) and frequency of proportions (**c**) for surface lake sediments. **d**, Spatial variation of coarse silt percentages (blue line) identified in surface lake sediments compared to the spatial variation of the mean grain-size (red line) of the corresponding sediments.

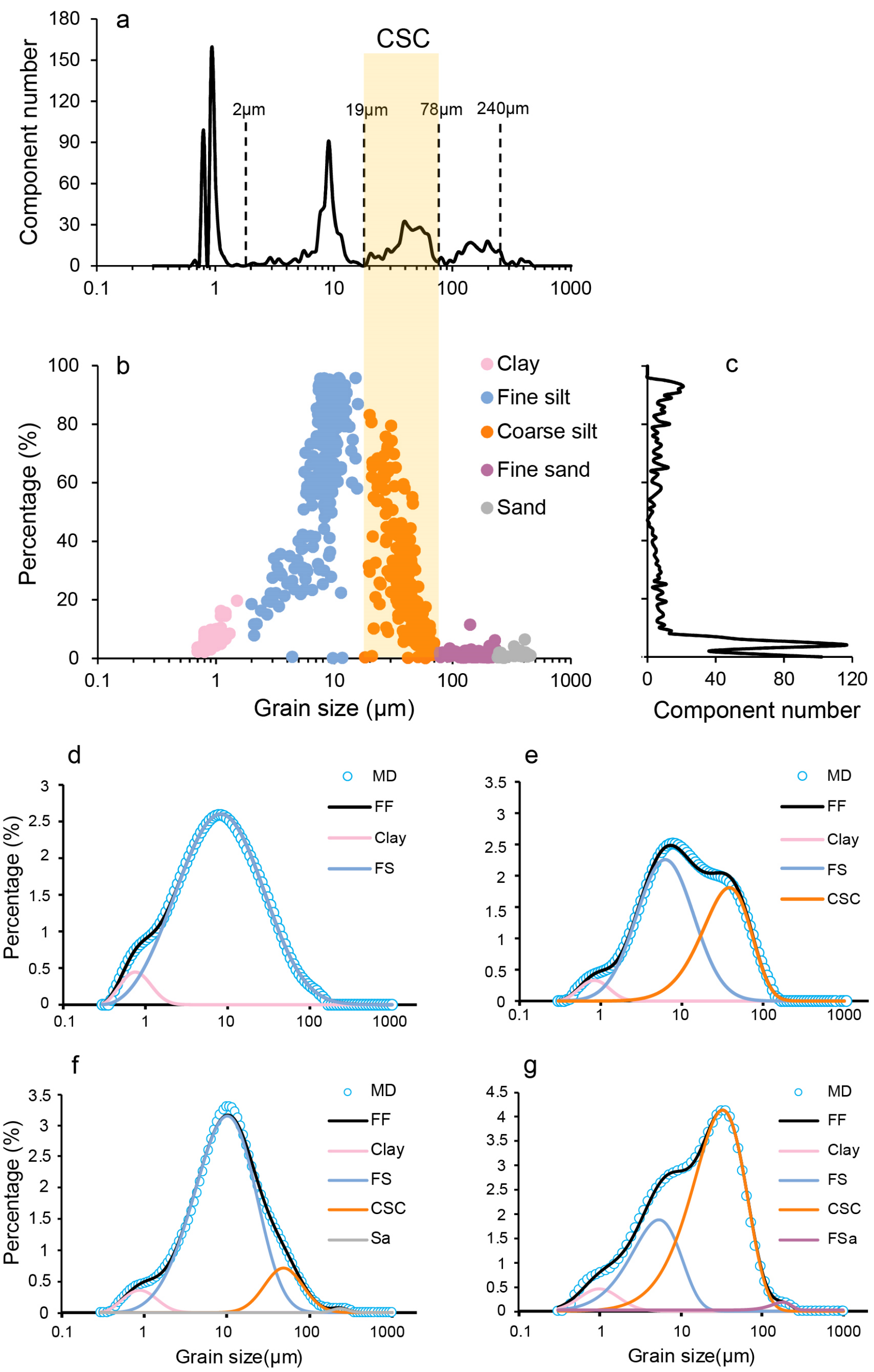

Supplementary Figure 4. Grain-size analysis results for sediments from long core GH09B. a-c, Frequency of modal sizes (**a**), size-proportion plot of all grain-size components (**b**) and frequency of proportions (**c**) of sediments of core GH09B. **d-g**, Grain-size analysis results of representative sediments of core GH09B. MD - measured data, FF - fitting function, FS - fine silt component, CSC - course silt component, FSa - fine sand component, Sa - sand component.

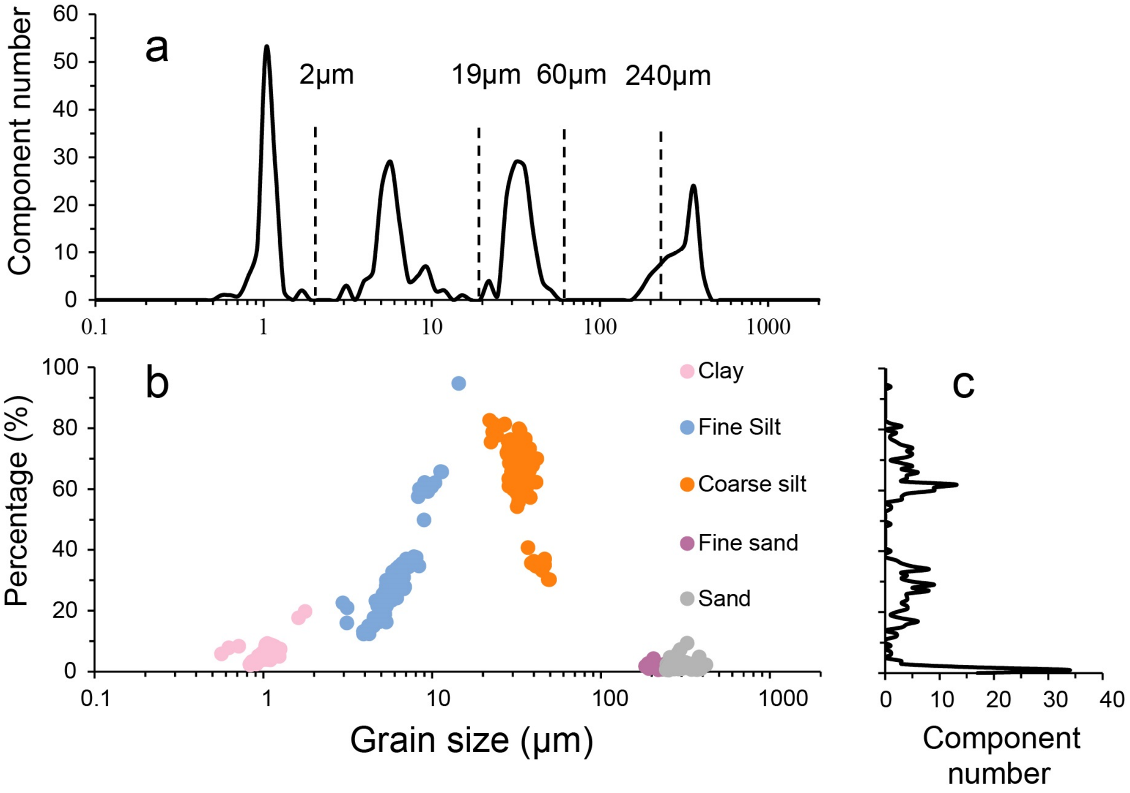

Supplementary Figure 5. Grain-size analysis results for sediments from short core GH13F. **a-c**, Frequency of modal sizes (**a**), size-proportion plot of all grain-size components (**b**) and frequency of proportion (**c**) of sediments of core GH13F.

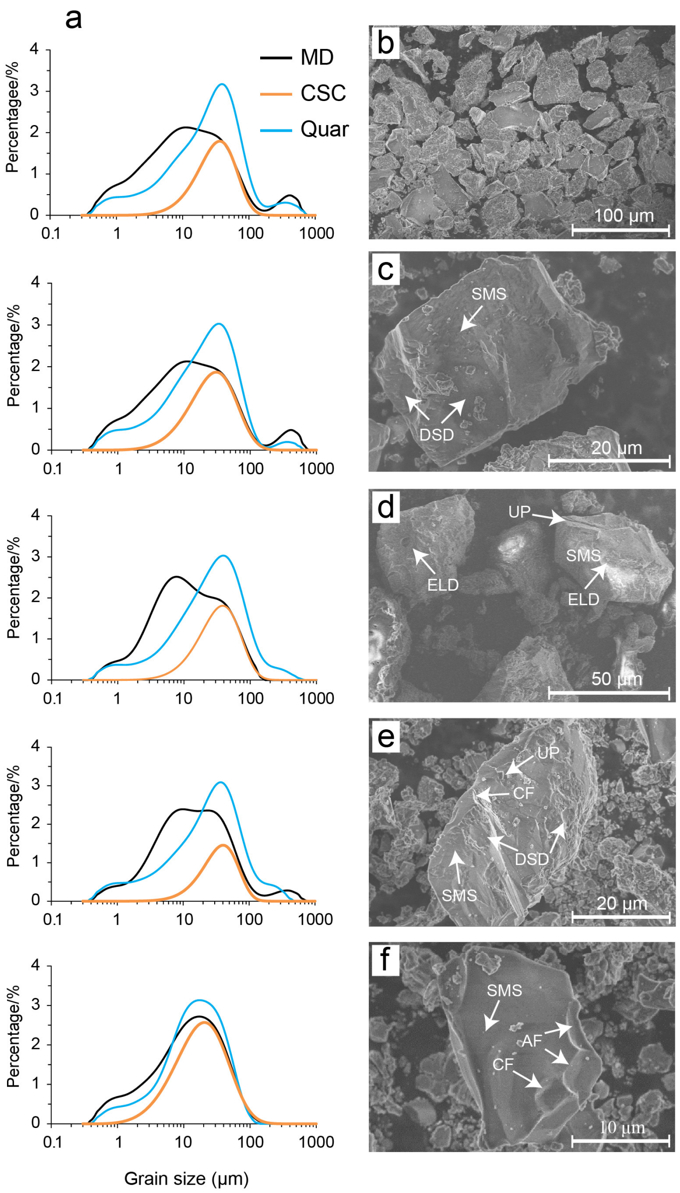

Supplementary Figure 6. Grain-size distributions and micromorphology of isolated quartz particles from sediments of core GH09B. a, Comparison of grain-size distributions of the CSC in the sediments of core GH09B with grain-size distributions of isolated quartz particles from the same sediments. MD: measured data, Quar: isolated quartz, CSC: coarse silt component. **b**, Scanning Electron Microscope (SEM) images of the clastic component showing sub-angular grains with different sizes. **c-f**, SEM images showing dish-shaped depressions (DSD; **c**, **e**, **f**), smooth precipitation surfaces (SMS; **c**, **d**, **e**, **f**), upturned plates (UP; **d**, **e**), elongated depressions (ELD; **d**), cleavage faces (CF; **e**, **f**), and arcuate fractures (AF; **f**).

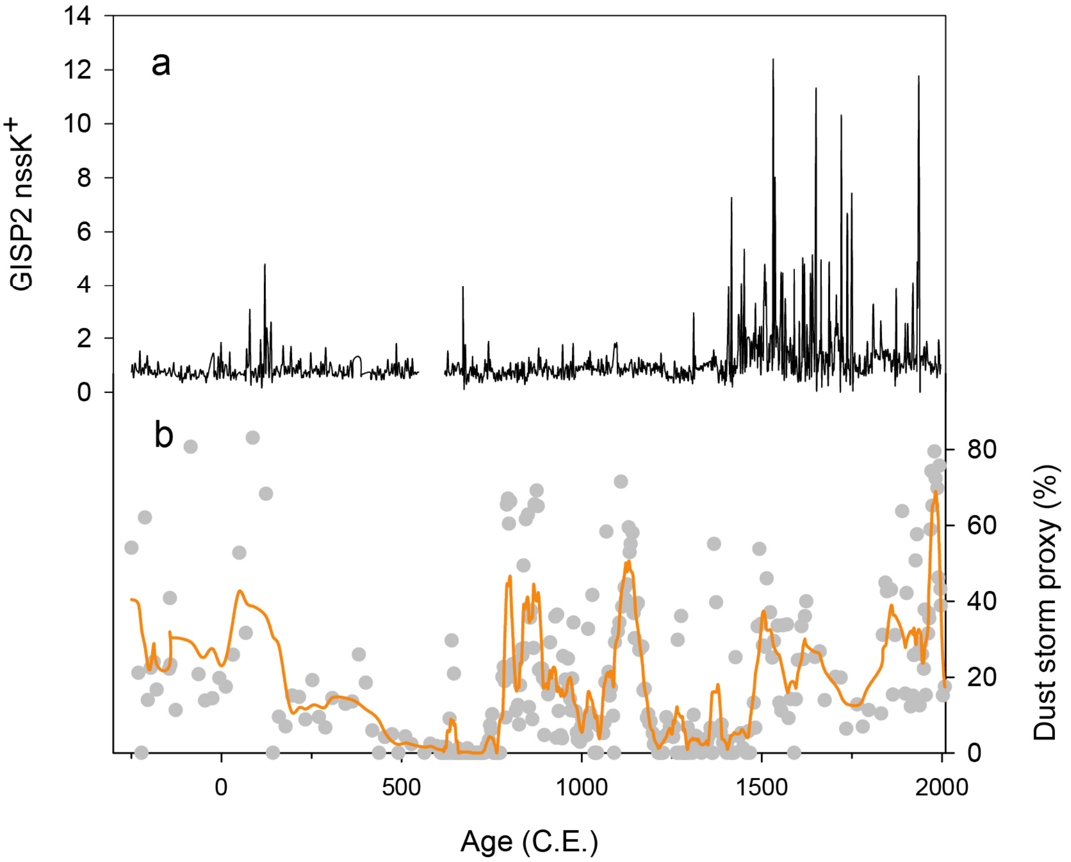

Supplementary Figure 7. Comparison of reconstructed dust storm activity and wind strength. a, Non-sea salt ions (nssK+) recorded in the GISP2 Greenland ice core, indicating changes in the intensity of the Siberian High[2]. **b**, Reconstructed dust storm record from Lake Gonghai (this study). The wind strength in northern China is closely associated with the intensity of Siberian High[3]. The comparison between these two records illustrate the limited role of wind strength in dust storm activity.

Lab ID	Sample No.	Depth (m)	Material	$\delta^{13}C$ ‰	Conventional Age (1σ, BP yr)	Calibrated Age (1σ, Cal yr BP)
Beta306751	GHB1-35	0.46	Stem	-25.9	150±30	0-282
XA4667	GHB1-48	0.63	Leaf	-24.9	368±23	332-498
XA5587	GHB1-82	1.07	Leaf	-15.7	570±25	540-628
XA4669	GHB1-89	1.16	Stem	-30.7	665±34	565-668
XA4670	GHB1-153	1.99	Stem	-30.9	1005±40	804-963
XA4668	GHB1-191	2.49	Stem	-30.4	1102±30	968-1053
XA4671	GHB2-76	3.63	Stem	-25.4	1329±25	1194-1294
XA5588	GHB2-112	4.06	Leaf	-28.7	2231±35	2159-2325

Supplementary Table 1. Radiocarbon dating results of terrestrial plant macrofossils from the uppermost 4.15 m of core GH09B from Lake Gonghai.

Supplementary Note 1

Interpretation of decreased dust storm activity during the early Qing Dynasty. In the early stage of the Qing Dynasty, farmers were prohibited from cultivating lands in the northern semi-arid region of China[4]. The restriction was initially alleviated at 1697 C.E., and after 1730 C.E. a large number of farmers started to migrate to the region and reclaim land[4]. The timing of the prohibition is consistent with an interval of low dust storm activity, which explains why dust storm activity decreased even though there was an increasing population after the establishment of the Qing Dynasty.

Supplementary References

1. Sun, D. H., Chen, F. H., Bloemendal, J. & Su, R. X. Seasonal variability of modern dust over the Loess Plateau of China. *J. Geophys. Res.* **108**, 4665 (2003).
2. Mayewski, P. A. & Maasch, K. A. Recent warming inconsistent with natural association between temperature and atmospheric circulation over the last 2000 years. *Clim. Past Discuss.* **2**, 327–355 (2006).
3. M. M. Yoshino, *Climatic Change and Food Production* (University of Tokoy, Tokoy, 1978), pp. 331.
4. Wang, S. Y. Historical changes of pastoral and farming economy in Ordos Plateau and its impacts on natural environment. *Hist. Geogr.* **5**, 11–24 (1985).
